# Coordinated Cytokine Responses Are Associated With Rapid HIV Reservoir Decay During Acute Treated HIV Infection

**DOI:** 10.64898/2026.08.18.26360728

**Published:** 2026-10-24

**Authors:** Alton Barbehenn, Erik Lundgren, Sonia Savur, Lei Shi, Junzhe Shao, Rebecca Hoh, Heather M. Hartig, Vivian Pae, Sannidhi Sarvadhavabhatla, Sophia Donaire, Caroline H. Sheikhzadeh, Jeffrey Milush, Gregory M. Laird, Mignot Mathias, Kristen Ritter, Jeffrey Martin, Frederick Hecht, Christopher Pilcher, Stephanie E. Cohen, Susan Buchbinder, Diane Havlir, Monica Gandhi, Timothy J. Henrich, Hiroyu Hatano, Susan P. Ribeiro, Jeffrey A. Tomalka, Steven G. Deeks, Rafick P. Sekaly, Jingshen Wang, Aaron Hudson, Sulggi A. Lee

## Abstract

**Background:**

The HIV reservoir is established within days of infection and persists despite antiretroviral therapy (ART). Host immune responses associated with early reservoir decay remain poorly defined.

**Methods:**

We analyzed > 500 longitudinal blood samples from 67 individuals treated during acute HIV infection. Plasma cytokines and HIV reservoir size (intact and defective DNA) were quantified longitudinally. Cytokine–reservoir relationships were assessed using unsupervised clustering and complementary mixed-effects nonlinear and linear spline models; counterfactual modeling examined how cytokine concentrations at defined timepoints related to subsequent reservoir trajectories.

**Results:**

TNF-α, IL-17A, and IL-10 were significantly associated with intact HIV DNA during the first 24 weeks of ART after false-discovery-rate correction. TNF-α showed one of the strongest associations with intact reservoir decay, whereas IL-10 showed the most consistent association across intact and defective HIV DNA. A coordinated cytokine cluster comprising IFN-γ, IL-10, IL-18, and TNF-α was associated with both reservoir measures. Phase-specific and counterfactual analyses further suggested that associations between several cytokines and reservoir dynamics varied with timing during early ART. These relationships persisted after adjustment for ART initiation timing, baseline viremia, initial CD4+ T-cell count, and longitudinal CD4:CD8 ratio.

**Discussion:**

Coordinated inflammatory, antiviral, and immunoregulatory cytokine responses are associated with HIV reservoir decay following ART initiation during acute infection, with some relationships varying over time. These findings may inform future stage-specific HIV cure strategies that leverage the timing and balance of antiviral, inflammatory, and immunoregulatory pathways to limit reservoir persistence.

**Main Summary:**

In people initiating ART during acute HIV infection, coordinated inflammatory, antiviral, and immunoregulatory cytokine responses were associated with faster HIV reservoir decay, with TNF-α and IL-10 showing particularly strong and consistent associations.

